# Immunometabolic crossroads: infections as bidirectional modulators in diabetes and metabolic syndromes

**DOI:** 10.3389/fendo.2025.1710157

**Published:** 2025-12-01

**Authors:** Md. Sharifull Islam, Sawda Binte Monir, Nabila Haque, Marshia Ahmed Vabna, Jie Fan, Yikui Li, Ishatur Nime, Farahnaaz Feroz, Mrityunjoy Acharjee, Fan Pan

**Affiliations:** 1 Center for Cancer Immunology, Institute of Biomedicine and Biotechnology, Shenzhen Institute of Advanced Technology (SIAT), Chinese Academy of Sciences (CAS), Shenzhen, China; 2 Department of Microbiology, Stamford University Bangladesh, Dhaka, Bangladesh; 3 Department of Microbiology, Jahangirnagar University, Dhaka, Bangladesh; 4 Key Department of Pathology, School of Basic Medicine, Henan University of Science and Technology, Luoyang, China; 5 Key Laboratory of Environment Correlative Dietology, College of Food Science and Technology, Huazhong Agricultural University, Wuhan, Hubei, China

**Keywords:** metabolic dysregulation, insulin resistance, infections and diabetes, chronic inflammation, therapeutic strategies

## Abstract

Diabetes and metabolic disorders represent a global health crisis driven by complex interactions between metabolic, immune, and microbial networks. Beyond their metabolic derangements- hyperglycemia, insulin resistance, and low-grade systemic inflammation-these disorders are now recognized to exist at an immunometabolic interface profoundly influenced by infectious agent The bidirectional relationship between infections and metabolic dysregulation highlighting how acute and chronic infections contribute to insulin resistance, β-cell dysfunction, and systemic inflammation, while metabolic dysregulation impairs immune competence, predisposing individuals to recurrent and severe infections. Pathogens such as *Helicobacter pylori Staphylococcus aureus*, *Escherichia coli*, SARS-CoV-2, and hepatitis viruses, alter host metabolic signaling through inflammatory, mitochondrial, and hormonal pathways, reshaping glucose and lipid homeostasis. In turn, diabetic immune impairment amplifies susceptibility to pneumonia, urinary tract infections, and chronic wound infections, reinforcing a pathogenic feedback loop. Emerging therapeutic strategies including nanotechnology enabled, therapeutics, gene, and stem cell based interventions and next-generation incretin agonists- including tirzepatide and CagriSem offer promising avenues to restore both metabolic balance and immune resilience. Additionally, foundational strategies such as lifestyle modifications, medical nutrition therapy, and vaccination remain essential components of disease control. Understanding infections as dynamic modulators of metabolic homeostasis reframes diabetes not merely as an endocrine disorder, but as a systemic immunometabolic disease. This review synthesizes current evidence on infection induced metabolic syndrome, immune impairments, and innovative therapeutic strategies to guide future precision interventions at the infection-metabolism interface.

## 1 Introduction

Diabetes is a multifaceted condition that fundamentally disrupts metabolic homeostasis. It often begins with subtle symptoms like excessive thirst, polyuria, and fatigue- but conceals a complex pathophysiological network (1). At its core lies an imbalance in insulin production or action, leading to sustained hyperglycemia and progressive multi-organ dysfunction. Type 1 diabetes arises from autoimmune destruction of pancreatic beta-cells, whereas type 2 diabetes (T2DM), the predominant form, evolves gradually through lifestyle and weight associated insulin resistance (2). Notably, adipose tissue functions as an active endocrine organ, secreting cytokines and adipokines that sustain chronic inflammation and impair insulin signaling. This low-grade inflammatory state damages vasculature and organ systems, further amplifying metabolic dysregulation (3). Neural circuits governing appetite and energy expenditure also play a critical role, and their disruption reinforces the cycle of weight gain and insulin resistance. Beyond biology, social determinants—including nutrition, healthcare access, and education—profoundly shape disease onset and progression (4, 5). Metabolic disorders such as obesity, T2DM, and metabolic syndrome (MetS) share overlapping pathophysiological features: hyperglycemia, dyslipidemia, hypertension, visceral adiposity, and endothelial dysfunction. Chronic inflammation is a central driver, mediated by immune cells including CD11b^+^CD11c^+^ myeloid cells and CD3^+^ T cells, and cytokines such as interleukin-1β (IL-1β), tumor necrosis factor-α (TNF-α), and interleukin-6 (IL-6) (6, 7). These pathways are not unique to metabolic disease but echo mechanisms observed in rheumatoid arthritis, atherosclerosis, and other inflammatory conditions, underscoring a shared immunometabolic architecture.

Infections—both acute and chronic—are increasingly recognized as modulators of this architecture. Acute infections provoke systemic inflammation and transient insulin resistance through cytokine and stress-hormone cascades (8), while chronic infections sustain inflammatory signaling, disrupt β-cell integrity, and induce gut dysbiosis that perpetuates metabolic dysfunction (9).

Pathogens including Helicobacter pylori, SARS-CoV-2, Salmonella, and cytomegalovirus (CMV) have been linked to obesity, MetS, and T2DM through inflammatory, mitochondrial, and microbiome-mediated pathways (10–13).

Interestingly, certain helminth infections, such as *Schistosoma mansoni*, may confer metabolic protection by rebalancing immune responses. Understanding these bidirectional links between infection and metabolism is essential for refining prevention and treatment strategies. This review synthesizes emerging evidence on how infections perturb immunometabolic networks, explores diagnostic and therapeutic challenges, and highlights translational opportunities to harness infection–metabolism interactions for improved clinical outcomes.

## 2 Methods

### 2.1 Search strategy

We have conducted a comprehensive search across multiple databases, including PubMed, Embase, and Web of Science from January 2000 to July 2025, to identify eligible studies. The search strategy combined MESH (Medline) and free terms using the Boolean operators “AND” and “OR”. “Infections and diabetes”, “metabolic dysregulation”, “Chronic inflammation” and “therapeutic strategies” were terms used in the search. A complementary search was carried out in the references of studies included.

#### 2.1.1 Inclusion criteria

All original peer reviewed research publications were considered. Eligible studies included observational human studies specifically examining infection, inflammation and metabolic disorders in diabetic patients compared with control groups.

#### 2.1.2 Exclusion criteria

Papers with incomplete or insufficient data or reporting information are excluded. The non-English studies; studies with only abstracts available; and studies with high risk of bias were eliminated.

## 3 Evolving landscape of diabetes

Despite remarkable advances in medical science, diabetes remains a difficult condition to manage. Novel pharmacological agents continue to emerge, targeting specific disease mechanisms such as enhancing insulin sensitivity or preserving pancreatic β-cell function (14). Yet, lifestyle modification- through healthy eating and regular physical activity- remains the cornerstone of therapy. Researchers are increasingly exploring personalized treatment strategies, with genetic profiling expected to refine therapeutic precision in the future. Diabetes is often described as a silent disease that gradually reconfigures the body’s physiological balance and quality of life (15). Understanding diabetes therefore requires looking beyond glycemic indices to appreciate its broad, systemic influence on overall health. With ongoing research and individualized care, there is cautious optimism for improved disease control and enhanced quality of life (16). The prevailing perspective has shifted: diabetes is no longer viewed as a straightforward metabolic disorder with predictable complications (17). It is now recognized as a complex, multisystem condition that subtly but profoundly alters organ function. This paradigm shift urges clinicians to screen for a wider spectrum of complications and challenges researchers to move beyond glucose-centric approaches in pursuit of more effective, mechanism-based therapies (18).

## 4 Infection and inflammation: interlinked but distinct mechanisms

Infection and inflammation are distinct biological processes that are closely associated in most of the diseases (19). Infection is the invasion and multiplication of pathogens in the host tissue (20), which can cause tissue injury and elicit a protective immune response (21). In contrast, inflammation is a highly coordinated response of the body to harmful triggers, such as microorganisms, irritants or destroyed cells. It is not the same as infection but usually accompanies it. Infection may trigger inflammation, yet it also arises under sterile circumstances, like in autoimmune and metabolic disorders (22). In chronic diseases like periodontitis, an excessive inflammatory response can become more detrimental than the pathogens themselves (23). Chronic inflammation is characterized by persistent immune stimulation, oxidative stress, and sustained cytokine release, all of which contribute to ongoing tissue injury and systemic complications (24). Thus, although infection initiates immune activation, dysregulated or persistent inflammation drives disease progression and severity. This interplay is particularly evident in diabetes, where inflammation is intricately involved in both disease onset and amplification of pathological changes (25).

## 5 Infection susceptibility and immune dysfunction in diabetes

Diabetes progressively reconfigures the body’s internal defense mechanisms far beyond hyperglycemia. What begins as a metabolic disorder gradually impairs immune responses, induces chronic low-grade inflammation, and heightens vulnerability to infections that are more frequent, severe, and difficult to treat (26). These processes form a self-perpetuating cycle in which metabolic dysfunction, immune impairment, and inflammation exacerbate one another (27). Neutrophils and macrophages for instance exhibit delayed migration, impaired phagocytosis, and reduced bactericidal activity, leading to slower clearance of pathogens (28). Consequently, individuals with diabetes are more susceptible to persistent infections such as urinary tract infections (UTIs), pneumonia, and skin or soft-tissue infections (29). Their recovery prospects are often poorer compared with non-diabetic individuals.

Beyond immune suppression, diabetes fosters persistent, mild inflammatory responses driven by metabolic stress rather than infection or injury (30). Excess glucose, oxidized lipids, and toxic by-products trigger inflammatory cascades that upregulate cytokines such as IL-6 and TNF-α (31, 32).

Over time, this chronic inflammation damages vasculature and connective tissue, compromising barrier integrity, delaying wound healing, and facilitating microbial invasion.

Over time, this chronic inflammation damages vasculature and connective tissue, compromising barrier integrity, delaying wound healing, and facilitating microbial invasion (33). Consequently, even minor infections can escalate rapidly into life-threatening complications.

The consequence is that even minor infections may rapidly escalate into life-threatening complications. These insights shift the perception of diabetes from a condition centered solely on elevated blood sugar to a systemic disorder that fosters an environment conducive to persistent infection, impaired healing, and unrelenting inflammation (34). Effective diabetes management requires more than glycemic control. A comprehensive strategy must address inflammatory regulation, immune system reactivation, judicious antibiotic use, and, where appropriate prophylactic measures such as vaccination to prevent infection-related complications (35, 36). **Table 1** summarizes the most common bacterial infections associated with diabetes, outlining the causative pathogens, clinical impacts, and primary risk factors- underscoring the need for a multifaceted therapeutic approach.

**Table 1:** Common bacterial infections associated with diabetes and their clinical implications.

Infection	Common pathogens	Effect of diabetes	Risk factors in diabetes	References
Conjunctivitis	*Staphylococcus epidermidis, Pseudomonas* sp.	Weakened ocular surface barrier	Chronic hyperglycemia	(37, 38)
Malignant External Otitis	*Pseudomonas aeruginosa*	Can progress to skull base osteomyelitis	Poor glycemic control	(39, 40)
Urinary Tract Infections (UTIs)	*Escherichia coli*, *Klebsiella* spp.	Can worsen glycemic control; recurrent infections common	Poor bladder emptying, immune dysfunction, glucosuria	(41–43)
Pneumonia	*Streptococcus pneumoniae*, *Haemophilus influenzae*	Increased severity and hospitalization rates	Impaired mucociliary clearance, immune suppression	(44)
Periodontal Infections	*Porphyromonas gingivalis*, *Prevotella* spp.	Exacerbate systemic inflammation and insulin resistance	Poor oral hygiene, altered host immune response	(45, 46)
Tuberculosis (TB)	*Mycobacterium tuberculosis*	Bidirectional: TB worsens glucose control, and diabetes increases TB risk	Immunosuppression, crowded living conditions	(29, 47)
Bacterial Sepsis	*E. coli*, *Klebsiella* spp., *S. aureus*	Higher ICU mortality, organ dysfunction	Delayed treatment, immune dysregulation	(48, 49)
Foot and Soft Tissue Infections	*Staphylococcus aureus*, *Streptococcus* spp.	Can lead to ulcers, gangrene, amputations	Peripheral neuropathy, poor circulation, poor wound healing	(49, 50)
Skin and Subcutaneous Abscesses	*S. aureus* (including MRSA)	Slower healing, higher recurrence	Hyperglycemia, obesity, poor hygiene	(51, 52)
Surgical Site Infections (SSIs)	*S. aureus, Enterococcus* spp.	Slower healing, increased post-op complications	Poor pre-op glucose control, immunocompromise	(53, 54)

**Figure 1:**
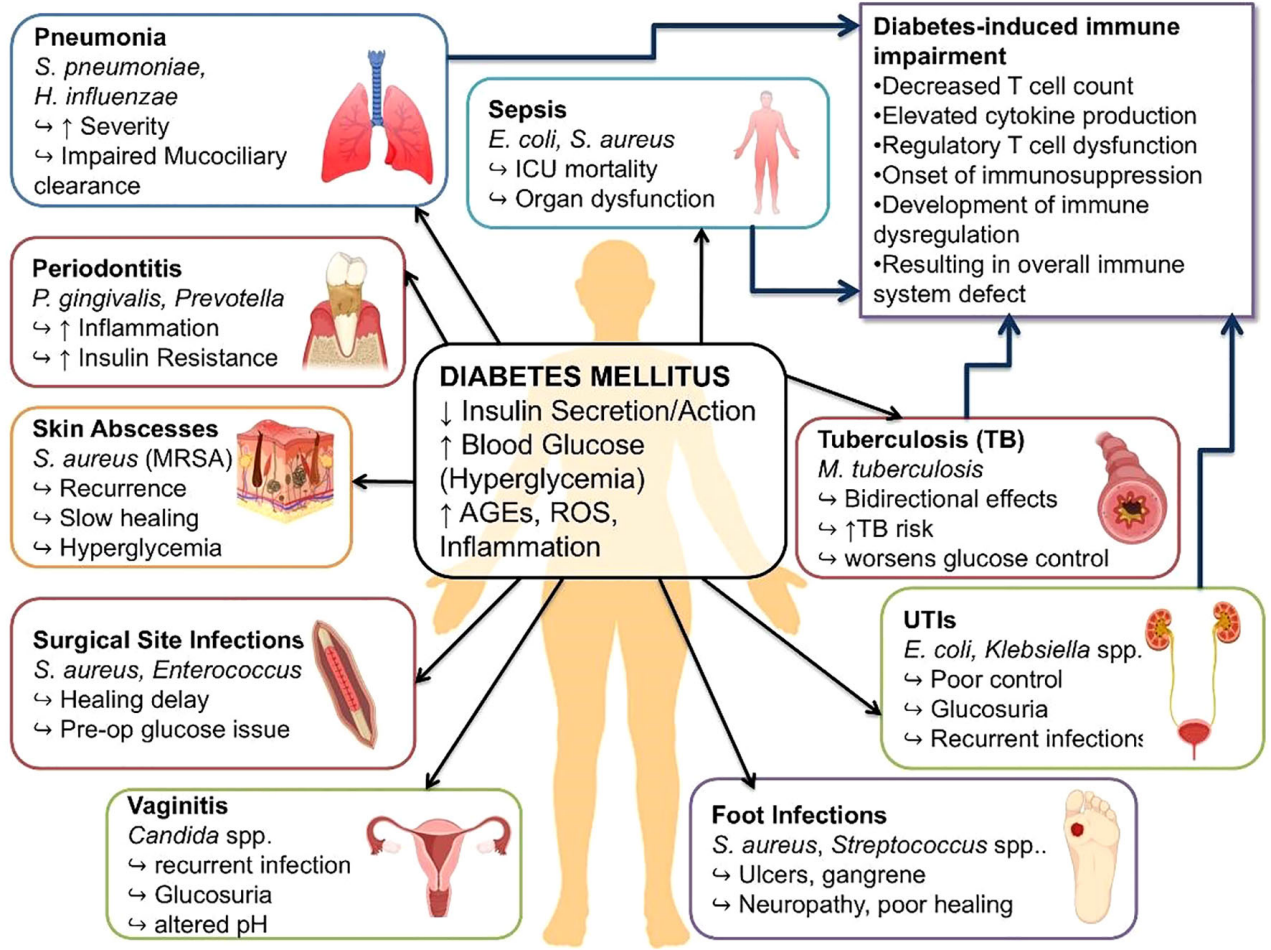
Organ-specific bacterial infections in diabetes: pathogens, mechanisms, and immune impairments. This figure illustrates the central metabolic conditions, including oxidative stress, resistance towards insulin, increase in Reactive Oxygen Species (ROS) and increase in (AGEs) Advanced Glycation End products (compounds of lipid or protein after exposure to sugar), which decrease immunological defenses. Individuals with diabetes exhibit heightened vulnerability to urinary tract infections (UTIs), pneumonia, tuberculosis (TB), sepsis, periodontitis, skin abscesses, surgical site infections, and diabetic foot infections, as well as less commonly addressed yet equally consequential complications such as keratitis, endophthalmitis, and malignant otitis externa.

People with diabetes are more susceptible to infections because of compromised immune function. Common infections include urinary tract infections (UTIs), skin and soft tissue infections, and respiratory tract infections (**Figure 1**). Less common but severe infections like malignant otitis externa as well as necrotizing fasciitis, both linked to hyperglycemia (55). UTIs are particularly frequent and persistent due to glucosuria, incomplete bladder emptying, and impaired neutrophil activity (56). Diabetic individuals also exhibit reduced mucociliary clearance and defective alveolar macrophage function, predisposing them to more severe forms of pneumonia (57, 58). Susceptibility to *Mycobacterium tuberculosis* infection is increased due to diminished T cell-mediated immunity, creating a vicious cycle in which tuberculosis further worsens glycemic control (59). Periodontal infections are more severe and chronic and are fueled by a dysregulated neutrophil response and pro-inflammatory gingival milieu, and can further potentiate insulin resistance (60). Recurrent skin abscesses are also common, typically resulting from poor glycemic control and impaired polymorphonuclear leukocyte function, frequently involving *Staphylococcus aureus*, including methicillin-resistant strains (MRSA) (61, 62). Foot and soft-tissue infections, often progressing to osteomyelitis or gangrene, arise due to peripheral neuropathy, poor vascular perfusion, and defective leukocyte function (63). Wound infections are more common in patients with inadequate pre-operative glucose control, resulting in delayed granulation and complications (64). Diabetic sepsis is characterized by an unbalanced immune response and a higher mortality rate mostly due to *E. coli, Klebsiella*, and *S. aureus* (65, 66). Hyperglycemia impairs key neutrophil functions, including chemotaxis and phagocytosis, further reducing infection clearance (67). Neuropathy and vascular injury in diabetes compromise skin integrity and disrupt mucosal barriers, facilitating bacterial invasion (68, 69). Consequently, pathogenic organisms such as *Klebsiella pneumoniae* and *Burkholderia pseudomallei* frequently cause liver abscesses and other severe infections in diabetic patients (70).

The most prevalent impact of diabetes on the eyes is a condition called diabetic retinopathy, but it also raises the possibility of non-retinal ocular infection (71). Bacterial keratitis and conjunctivitis are much more common and severe and recover more slowly in diabetic patients (72). Poor glycemic control compromises the ocular surface and local immune defense, increasing vulnerability to bacterial infections caused by *Staphylococcus aureus* and *Pseudomonas aeruginosa* (73). Beyond these, microvascular and neuropathic damage predisposes patients to serious infections such as orbital cellulitis (74). Moreover, postoperative endophthalmitis occurs more frequently in diabetic individuals after cataract surgery, driven by delayed wound healing and dysregulated immune responses (75, 76). Surgical site infections (SSIs) also occur more frequently in diabetic patients largely due to hyperglycemia-induced impairments in leukocyte function and delayed tissue repair (77). Poor preoperative glycemic control significantly increases the risk of SSIs, especially following abdominal or orthopedic surgeries. These infections often involve multidrug-resistant organisms such as *S. aureus* and *Enterococcus*, and are further aggravated by comorbidities like obesity and peripheral arterial disease, leading to prolonged recovery, higher morbidity, and increased healthcare burden (78). Diabetes significantly elevates hospitalization and mortality following infection; one cohort study found that it doubled the risk of sepsis and tuberculosis admissions (47). These adverse outcomes are linked to delayed diagnosis, comorbidities, and suboptimal glycemic control. Diabetic individuals also show increased antimicrobial resistance, complicating management (79). Optimal glycemic regulation remains the most effective approach to reduce infection risk and enhance immune resilience (80). Preventive measures—such as vaccination against *Streptococcus pneumoniae* and influenza—and education on foot hygiene and early infection recognition are critical (81, 82). A multidisciplinary management strategy integrating endocrinology and infectious disease expertise can substantially improve outcomes.

## 6 Interactions between metabolic disorders and inflammation

**Figure 2:**
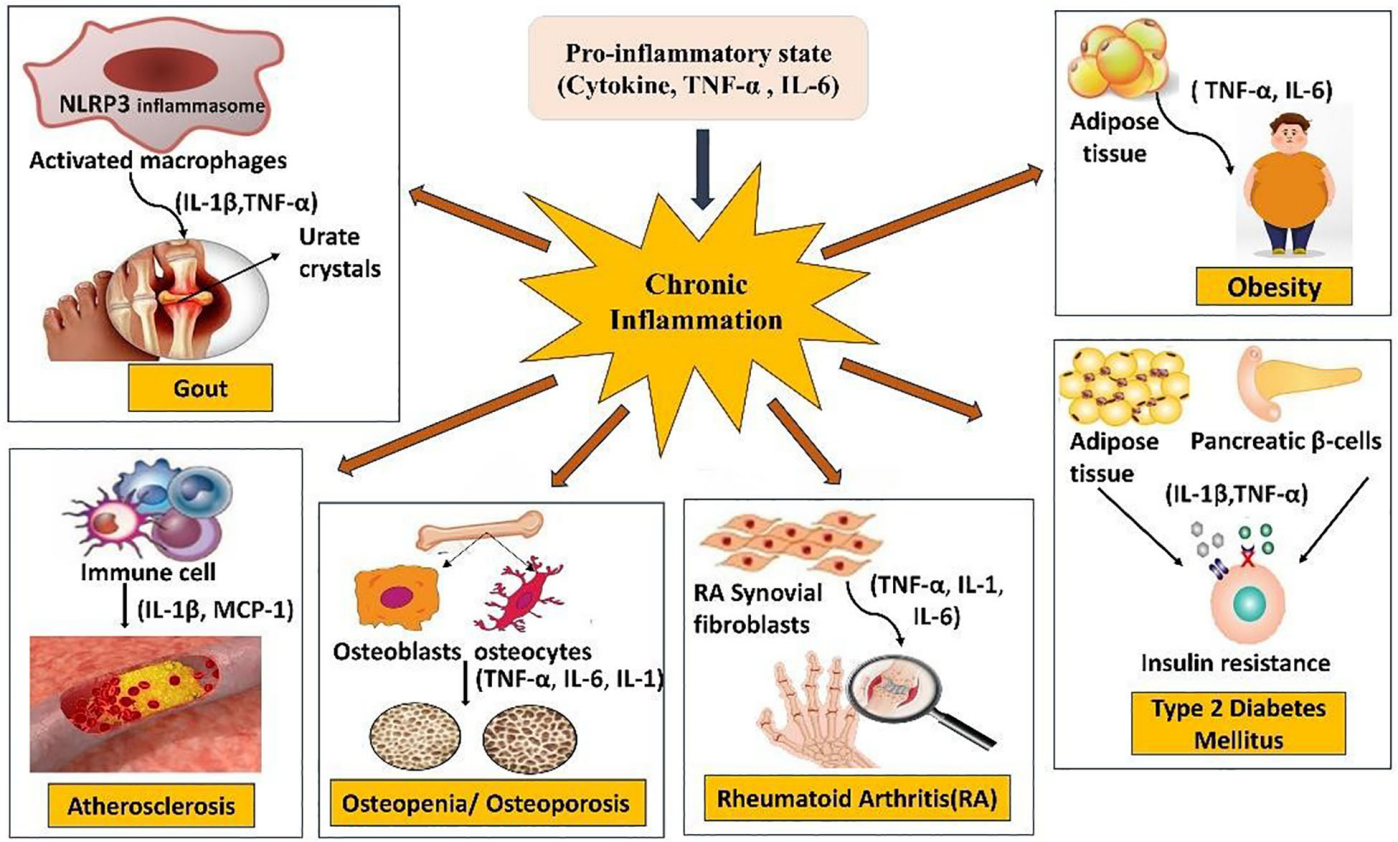
Cross talk between inflammation and metabolic disorders. This illustration demonstrates how chronic inflammation contributes to a number of disorders. It is defined by a pro-inflammatory condition with elevated cytokines like interleukin-1 beta (IL-1β), interleukin-6 (IL-6), and tumor necrosis factor-alpha (TNF-α). By activating macrophages through the NLRP3 (NOD-, LRR-, and pyrin domain-containing protein 3) inflammasome, urate crystals in gout cause the release of TNF-α and IL-1β. Immune cells release Monocyte Chemoattractant Protein-1 (MCP-1) and IL-1β in atherosclerosis. Osteopenia/osteoporosis results from inflammatory cytokines acting on bone cells. Rheumatoid arthritis (RA) involves RA synovial fibroblasts producing pro-inflammatory cytokines. Type 2 diabetes mellitus (T2DM) and obesity are linked to inflammation in adipose tissue, which interferes with insulin signaling through IL-1β and TNF-α.

Metabolic diseases such as atherosclerosis, diabetes mellitus, obesity, gout, rheumatoid arthritis (RA), osteoporosis, and osteopenia frequently coexist with acute or chronic inflammatory processes (**Figure 2**). Mounting evidence highlights inflammation as both a driver and consequence of metabolic dysregulation, revealing the tight bidirectional link between immune and metabolic pathways (83). Type 2 diabetes exemplifies this crosstalk, functioning as a low-grade inflammatory state involving CD11b^+^CD11c^+^ myeloid cells and CD3^+^ T lymphocytes that promote insulin resistance (IR). Elevated cytokines, particularly IL-1β, impair pancreatic β-cell function, exacerbating hyperglycemia (83). In RA, persistent synovitis and systemic inflammation underlie disease pathology, and therapies targeting pro-inflammatory cytokines—such as DMARDs—ameliorate joint destruction and improve outcomes (7). Similarly, acute gouty arthritis results from macrophage and neutrophil activation in response to monosodium urate (MSU) crystals, driving IL-1β–mediated sterile inflammation (84). Furthermore, bone remodeling is influenced by systemic inflammation and metabolic factors. In kidney transplant recipients, elevated serum phosphorus, alkaline phosphatase levels, and glucocorticoid use are associated with bone loss. Lifestyle interventions targeting inflammation are recommended to help preserve bone health (85). Cytokines such as TNF-α, IL-6, and IL-1β promote inflammation and metabolic dysfunction, whereas adiponectin and IL-10 exert anti-inflammatory effects. Altered cytokine profiles have even been linked to metabolic parameters in conditions like anorexia nervosa, underscoring the immune- metabolic nexus. In type 1 diabetes (T1D), elevated circulating IL-6, IL-8, and IL-10 correlate with hyperglycemia and dyslipidemia, suggesting their potential as biomarkers for disease activity Immune cells, in particular, produce cytokines, which are tiny signaling proteins that modulate and control inflammation, hematopoiesis, and immunity. Certain cytokines, such as TNFα, IL-6, and IL-1β, as well as others that are anti-inflammatory, such as adiponectin and IL-10, influence immune responses in metabolic disorders. Alterations in cytokine profiles correlate with physiological parameters such as body water content in anorexia nervosa, demonstrating the complex immune-metabolic crosstalk. Several studies have reported that Type 1 diabetes (T1D) is accompanied by elevated levels of proinflammatory cytokines in patients. In one study, serum concentrations of IL-6, IL-8, IL-10, and vitamin D were measured in T1D patients and healthy controls using ELISA. The findings revealed significantly higher levels of IL-6, IL-8, IL-10, fasting glucose, HbA1c, and lipid abnormalities in T1D patients compared with controls. However elevated cytokine levels were associated with disease severity, suggesting their potential role as predictive biomarkers for T1D progression (86).

Macrophages serve as key regulators of inflammatory tone. Their polarization toward either pro-inflammatory (M1) or anti-inflammatory (M2) phenotypes determines metabolic outcomes. M2-promoting signals- such as GLP-1 receptor activation—confer protection against atherosclerosis, while miR-6869-5p–mediated regulation of protein tyrosine phosphatase receptor type O supports M2 polarization in gestational diabetes (87). Lifestyle, diet, and environmental factors compound these immune–metabolic interactions. Obesity predisposes to gout and amplifies inflammation through IL-1β and TNF-α signaling in adipose tissue, contributing to insulin resistance and cardiovascular risk. The metabolic syndrome- characterized by central obesity, dyslipidemia, hypertension, hypercoagulability, and chronic inflammation- constitutes a shared substrate linking diabetes, gout, and cardiovascular disease (88).

## 7 Infection and its role in metabolic disorders

Emerging evidence highlights infections as potent modulators of metabolic homeostasis, influencing the onset, trajectory, and complications of major metabolic disorders. Acute and chronic infections can act as primary triggers for metabolic dysregulation by altering host immune and biochemical pathways (89). Viral infections—including influenza and coronaviruses—reshape host gene expression and lipid metabolism to favor viral replication, while concurrently impairing immune responses and aggravating underlying metabolic dysfunction (89). Similarly, bacterial components such as lipopolysaccharides (LPS), translocated into the circulation via increased intestinal permeability, activate pro-inflammatory cascades that induce insulin resistance and promote metabolic syndrome (MetS) (9). Acute infections provoke a systemic stress response that disrupts glucose balance. Pro-inflammatory cytokines (IL-1, TNF-α) and counter-regulatory hormones (glucagon, growth hormone, catecholamines, glucocorticoids) antagonize insulin action by reducing peripheral glucose uptake and increasing hepatic gluconeogenesis, leading to transient or sustained insulin resistance. Certain viral infections—particularly enteroviruses such as Coxsackie B1/B4, mumps, rubella, and cytomegalovirus—have been linked to type 1 diabetes mellitus (T1DM) by triggering autoimmune destruction of pancreatic β-cells (26). Pathogens can further hijack host metabolic networks to support replication, thereby reprogramming glucose and lipid metabolism and predisposing hosts to metabolic disorders (90).

Chronic infections exert longer-term effects through persistent low-grade inflammation that impairs β-cell function and insulin signaling—hallmarks of type 2 diabetes. This inflammatory milieu accelerates the development of MetS-associated comorbidities, including cardiovascular disease and non-alcoholic fatty liver disease (91). Moreover, chronic infections disrupt gut microbiota homeostasis, leading to dysbiosis that amplifies systemic inflammation and insulin resistance, perpetuating a self-sustaining cycle of metabolic dysfunction (9). However, the pathophysiology and development of numerous metabolic disorders are significantly influenced by infections. Key discoveries from current research on the connection between infections and metabolic diseases are summarized in **Table 2**.

**Table 2:** Interplay between infectious diseases and metabolic syndromes.

Infection type	Associated metabolic disorder(s)	Key findings	Reference
*Helicobacter pylori (H. pylori)*	Metabolic Syndrome (MetS), Obesity, Diabetes	*H. pylori* infection is strongly correlated with MetS components such as hypertension, obesity, and insulin resistance. Chronic inflammation induced by *H. pylori* may contribute to the development and progression of MetS.	(10)
COVID-19	Type 2 Diabetes, Dyslipidemia	The total risk of developing newly diagnosed diabetes is increased by 1.46 times when infected with COVID-19. The infection may exacerbate metabolic dysfunction through inflammatory pathways.	(11)
Helminth Infections	Type 2 Diabetes, Metabolic Syndrome	Certain helminth infections, such as Schistosoma mansoni, are associated with improved metabolic outcomes, including lower fasting blood glucose and reduced prevalence of MetS.	(13)
*Salmonella* spp.	Diabetes	Infection with *Salmonella* species, including *S. typhi* and *S. paratyphi*, has been linked to alterations in immune responses and may influence the development of diabetes.	(92)
Cytomegalovirus (CMV)	Metabolic Dysfunction	Chronic CMV infection may contribute to immune senescence and metabolic dysfunction, potentially influencing the development of metabolic disorders.	(93)
Hepatitis C virus (HCV)	Type 2 Diabetes Mellitus (T2DM), Insulin Resistance, Atherosclerosis, Hepatic Steatosis	HCV promotes insulin resistance via impaired insulin signaling pathways (e.g., PP2A, SOCS-3, IRS), induces both viral and metabolic steatosis, and raises risk of fibrosis, atherosclerosis, and T2DM; meta-analysis confirms significantly higher T2DM incidence in chronic HCV patients	(94, 95)
Hepatitis B virus (HBV)	Metabolic Syndrome, Dyslipidemia, Fatty Liver	Chronic HBV may protect against metabolic syndrome but can worsen liver disease when combined with obesity or diabetes.	(96, 97)
Gut Dysbiosis/Microbiota Alterations	Obesity, T2DM, Non-Alcoholic Fatty Liver Disease (NAFLD), Metabolic Syndrome	Gut microbiota imbalance—such as a high Firmicutes/Bacteroidetes ratio and low *Faecalibacterium prausnitzii*—is associated with inflammation, insulin resistance, NAFLD, and T2DM. Probiotics, prebiotics, and FMT may help restore microbial balance and improve metabolic outcomes.	(98, 99)

## 8 Current advances in management of diabetes and metabolic disorders

**Figure 3:**
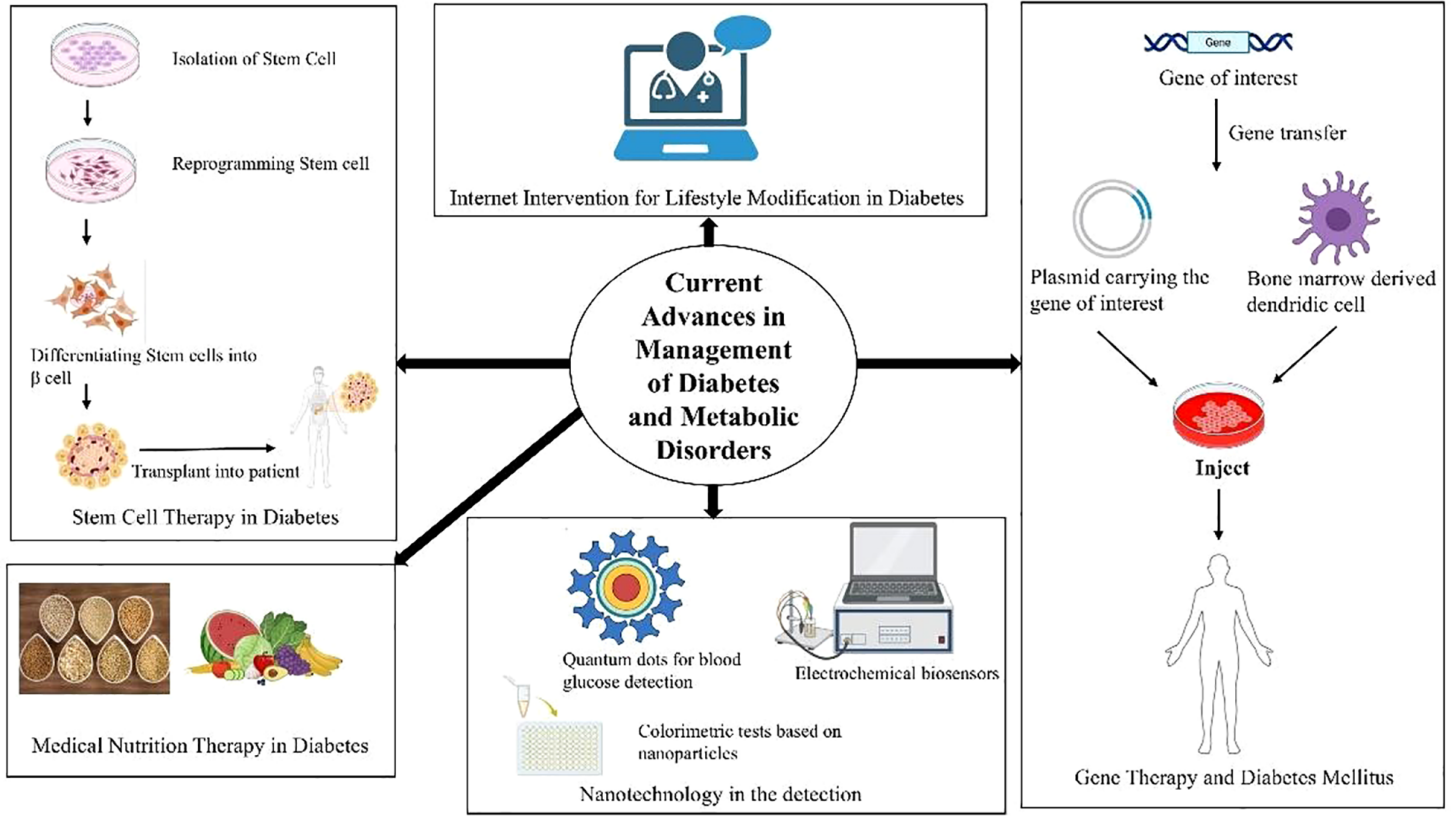
Current advances in management of diabetes and metabolic disorders.

Diabetes management has evolved through a combination of pharmacological innovation, lifestyle modification, and digital health integration. Despite these advances, early detection remains the cornerstone of successful disease control and prevention of complications (**Figure 3**) (100). The overarching therapeutic goal is to achieve optimal glycemic regulation while minimizing comorbid risks for each individual patient.

## 9 Internet-based interventions for lifestyle modification

Lifestyle modification remains a fundamental pillar in diabetes prevention and treatment. For individuals with prediabetes or established diabetes, sustained behavioral change- through increased physical activity, balanced nutrition, and cessation of smoking or excessive alcohol use- has consistently demonstrated metabolic benefit (101, 102).

While these strategies are effective for diabetes control, ensuring that individuals consistently follow them can be difficult. To support better adherence, internet-based tools and programs have been introduced. These digital platforms offer an accessible and effective means of promoting self-management among individuals with diabetes (103).

## 10 Nanotechnology and diabetes

Nanotechnology is redefining diabetes management by enabling non-invasive glucose monitoring, targeted insulin delivery, and next-generation cell- and gene-based therapies for Type 1 diabetes (T1DM). Its applications extend beyond treatment to early diagnosis, immune monitoring, and quantitative assessment of β-cell mass—critical since progressive β-cell loss underpins T1DM pathogenesis (104, 105). Traditional diagnostic tools often fail to detect diabetes at an early stage. Magnetic nanoparticles (MNPs), due to their unique properties, can be used in MRI to identify early beta-cell damage, allowing for timely intervention (105).

Accurate glucose tracking remains central to diabetes care, yet finger-prick testing is cumbersome and poorly adhered to. Although continuous glucose monitoring (CGM) systems using subcutaneous biosensors have improved glycemic control, they still face issues of sensor instability and frequent calibration (106, 107). Nanotechnology-enabled glucose sensors- utilizing functionalized nanoparticles such as glucose oxidase or glucose-binding proteins- offer superior sensitivity, faster response times, and greater patient convenience (100, 104).

Similarly, insulin therapy remains limited by injection discomfort, erratic absorption, and suboptimal glycemic regulation. Nanotechnology-based closed-loop delivery systems- so-called “nano-pumps”- integrate biosensing and feedback-controlled insulin release to achieve near-physiological glucose regulation (108, 109). Parallel research into oral, transdermal, and inhalable nanocarriers promises to transform insulin delivery into a minimally invasive, patient-friendly modality (108).

Beyond these clinical advantages, Nanoparticle-based systems can help reset the body’s metabolic–immune balance by rewiring how immune cells process energy, like flipping a switch inside each cell. For example, they can prompt macrophages to shift toward an oxidative, anti-inflammatory M2 profile rather than a glycolytic, proinflammatory M1 state, much like turning a dial from heat to cool air (110).

## 11 Medical nutrition therapy in diabetes

**Medical Nutrition Therapy (MNT)** represents a cornerstone of diabetes management, integrating individualized dietary assessment and intervention delivered by registered dietitian nutritionists. It targets the optimization of metabolic outcomes through tailored nutrition strategies and patient education (110, 111). International consensus groups have emphasized MNT’s growing importance as global dietary habits evolve, making structured nutritional intervention indispensable for both diabetes prevention and long-term glycemic control (110, 111).

MNT has shown particular efficacy in gestational diabetes mellitus (GDM), where carbohydrate intake critically influences maternal glycemia. The Institute of Medicine recommends a minimum intake of 175 g of carbohydrates per day during pregnancy, a target compatible with low-carbohydrate regimens traditionally employed in GDM management (112).

Clinical evidence supports that carefully balanced carbohydrate consumption can sustain euglycemia without adverse fetal outcomes (113). The central aim of MNT in GDM is to maintain optimal maternal blood glucose, support appropriate gestational weight gain, and prevent ketogenesis and metabolic acidosis (113).

Despite extensive research, the ideal macronutrient distribution for diabetes management remains undefined. Current data suggest that low-glycemic index (GI) diets are more beneficial than strict carbohydrate restriction for managing GDM, though confirmatory studies are needed (102, 113). Beyond pregnancy, calorie restriction remains a key strategy for individuals with overweight or obesity, supporting improved insulin sensitivity and weight reduction.

MNT’s strength lies in its individualization-designing dietary plans that align with medical needs, metabolic profiles, and lifestyle patterns. Collaborative models involving diabetologists and registered dietitians (RDs) are now being advanced to produce evidence-based nutritional frameworks that integrate MNT into routine diabetes care (114). Evidence indicates that MNT is a powerful, accessible, and cost-effective therapeutic approach that could play a vital role in diabetes prevention and care (111). Recent findings show that MNT can affect both blood sugar control and immune–metabolic pathways, with certain foods helping to regulate inflammation, curb oxidative stress, and shape immune cell activity through signals like AMPK, mTOR, and NF-κB (112).

## 12 Gene therapy and diabetes mellitus

Gene therapy represents a transformative approach with the potential to provide a one-time, long-term cure for numerous diseases, including diabetes mellitus (DM). Beyond simply inserting new genes, modern gene therapy encompasses gene modulation, editing, and silencing to correct or compensate for disease-causing abnormalities (114–116). In principle, it involves the therapeutic introduction, replacement, or suppression of genes within target cells to restore normal cellular function (117, 118). The field is broadly categorized into *somatic* and *germline* gene therapy. Somatic therapy targets non-reproductive body cells to correct patient-specific defects, whereas germline therapy modifies reproductive cells to prevent the hereditary transmission of genetic disorders (118).

The growing clinical interest in gene therapy stems from its potential to treat complex, multifactorial diseases such as diabetes, autoimmune disorders, cardiovascular diseases, and cancers—conditions that are often inadequately managed by conventional treatments (119). In the context of diabetes, particularly Type 1 diabetes mellitus (T1DM), gene therapy offers a novel approach to counteract autoimmune destruction of pancreatic β-cells, which is primarily driven by autoreactive T lymphocytes (120, 121). Since T1DM arises from a combination of genetic susceptibility and environmental triggers, identifying and targeting key genes involved in immune regulation and β-cell survival may offer curative potential (122).

(122).

Although current research predominantly focuses on T1DM, genetic factors also play significant roles in Type 2 diabetes mellitus (T2DM), opening the door for gene-based interventions in this subtype as well (123). Genome-wide association studies have identified more than 75 loci linked to T2DM pathogenesis, highlighting new therapeutic targets that influence both insulin sensitivity and β-cell function (121). Interestingly, these genetic loci appear to impact pharmacologic responsiveness more than disease onset itself, implying that gene-level modulation could refine personalized treatment strategies (124). For instance, inhibition of the *NLRP3* gene—known for driving inflammasome activation—has been shown in animal models to reduce pancreatic inflammation and apoptosis, thereby delaying or preventing T2DM development (124).

Overall, genes involved in Type 2 diabetes mellitus (T2DM) onset, progression, and complications represent viable targets for gene therapy. In the bigger picture, placing gene therapy within the context of immunometabolic modulation shows how it works to restore β−cell immune tolerance and steady metabolic balance, instead of acting as a lone genetic fix, much like tuning both the fuel and ignition in a finely running engine (125). **Table 3** summarizes genes that regulate glucose balance, enhance insulin production or sensitivity, and alleviate diabetes-related complications.

**Table 3:** Possible candidates that could be employed for gene therapy in type 2 diabetes mellitus.

Genes	Main function	Class	Ref
*GCK*	Controls insulin secretion stimulated by glucose and manages liver glucose metabolism.	Genes modulating homeostasis of glucose	(125)
*PPARG*	Plays a central role in adipocyte differentiation and glucose metabolism.		(126)
*GLUTS*	Engaged in the reuptake of filtered glucose from the kidneys back into the circulation.		(127)
*SGLTs*	Engage deeply in the hepatic and muscular glucose fluxes		(127)
*FGFs*	Plays a crucial role in maintaining the balance of glucose levels.		(127)
*SIRT6*	Associated with elevated glycolysis and GLUT expression		(127)
*LEPR*	Involved in appetite regulation and energy balance; leptin therapy improves insulin sensitivity.		(128)
*FOXO1*	Transcription factor regulating gluconeogenesis and β-cell function. Inhibition can improve insulin sensitivity.		(129)
*IRS1/IRS2*	Insulin receptor substrates critical in insulin signaling; enhance insulin sensitivity.		(130)
*GLP-1 and its analogs/agonists*	Increase beta-cell survival, stimulate insulin gene expression, and promote synthesis	Genes enhancing the secretion of insulin and/or sensitivity	(127)
*GPCRs and their agonists*	Increases the release of GLP-1 and insulin.		(127)
*CTB-APSL*	Boosts insulin secretion and contributes to insulin resistance.		(127)
*IKK E, TBK1*	Associated with a reduction in body weight, insulin resistance, fatty liver, and inflammation		(127)
*MAFA*	Transcription factor essential for insulin gene transcription in beta cells.		(129)
*GLIS3*	Regulates β-cell development and insulin gene transcription; mutations associated with neonatal diabetes.		(129)
*VEGFA*	Plays a role in diabetic retinopathy and microvascular repair.	Genes attenuating diabetic induced complications	(131)
*SOD1*	Attenuates oxidative stress-induced damage in diabetic tissues.		(132)
*IL-1b*	Associated with b-cell failure and inflammation		(127, 133)
*ADPN*	Reduces diabetic kidney damage		(127)
*TGF-a*	Has a role associated with nephron loss in DKD.		(127)
*NLRP3*	Reduces the severity of diabetic cardiomyopathy		(127)
*CDKN2A/2B*	Associated with the alteration of T-cell characteristics and persistent inflammation.		(127)
*HSP70*	Linked to the bioenergetics of mitochondria and diabetic sensory neuropathy		(127)
*MicroRNAs*	Involved in the regulation of microvasculature associated with diabetes		(127)
*TXNIP*	InInhibition reduces oxidative stress and beta-cell apoptosis		(132)

## 13 Stem cell therapy in diabetes

Traditional treatments for diabetes mellitus (DM) often fail to address the root causes and are frequently associated with adverse effects, prompting the search for alternative therapies. Currently, cellular-based approaches, such as pancreatic or islet-cell transplantation, aim to restore insulin secretion by replenishing beta cells. However, this method is limited by the scarcity of donor organs. Consequently, stem cell technology has gained attention as a promising solution to generate renewable sources of insulin-producing beta cells, potentially overcoming the donor shortage and providing long-term treatment options for DM patients (134). The pancreas is the organ of choice for regeneration research. Animal studies reveal that limited pancreatic tissue can regenerate optimal beta-cell mass, likely through replication and dedifferentiation of pancreatic ductal cells into pluripotent progenitors that subsequently generate new beta cells. These ductal cells can also be cultured *in vitro* to form insulin-producing clusters (128, 131). Experiments with mouse models demonstrated that bone marrow-derived progenitors could differentiate into functional beta cells ex vivo and that bone marrow cells could migrate to the pancreas, normalizing elevated blood glucose levels (135, 136). Clinical studies using autologous HSC transplantation have reported improvements in both Type 1 and Type 2 diabetes, highlighting their therapeutic potential (137, 138).Bringing stem−cell–based methods into the larger field of diabetes research shows how they can help restore β−cell function and steady the body’s metabolism, weaving regenerative treatments into the immune−metabolic processes driving the disease (139). Beyond stem cell advances, ongoing research continues to develop new pharmacological agents for diabetes management, with several drugs currently undergoing clinical trials and others recently approved for clinical use.

## 14 Current research on diabetes and metabolic syndrome

Alongside the advancements in diabetes management, a number of medications are currently in various phases of clinical trials for potential application. Additionally, some have completed the process and have been recently launched in the market.

## 15 Recently approved antidiabetic agents

### 15.1 Tirzepatide

Tirzepatide, recently approved by the U.S. Food and Drug Administration under the brand name *Mounjaro*, represents a paradigm shift in the pharmacological management of type 2 diabetes mellitus (T2DM) (138). Administered as a once-weekly subcutaneous injection, tirzepatide is the first dual incretin receptor agonist that simultaneously targets the glucagon-like peptide-1 (GLP-1) and glucose-dependent insulinotropic polypeptide (GIP) pathways—two central regulators of postprandial glucose homeostasis.

Mechanistically, GLP-1 enhances glucose-dependent insulin secretion, suppresses glucagon release, and delays gastric emptying, whereas GIP primarily augments insulin secretion under hyperglycemic conditions but may stimulate glucagon secretion during hypoglycemia (139). By co-activating both GLP-1 and GIP receptors, Tirzepatide amplifies insulinotropic and glucoregulatory effects while mitigating counter-regulatory hormonal fluctuations, thus achieving a more physiological balance of glycemic control (139).

(139).

The clinical efficacy of tirzepatide has been established through a series of global phase 3 SURPASS trials that benchmarked its performance against placebo, the GLP-1 receptor agonist semaglutide, and two basal insulin analogues—insulin degludec and insulin glargine—both as monotherapy and as adjunct therapy (140). Across these studies, tirzepatide achieved robust and dose-dependent reductions in glycated hemoglobin (HbA1c), with decreases of up to 1.5% in combination regimens and 11.6% as monotherapy relative to baseline. At its highest tested dose (15 mg), tirzepatide demonstrated superior efficacy, reducing HbA1c by approximately 0.5% more than semaglutide, 0.9% more than insulin degludec, and 1.0% more than insulin glargine (140).

Due to its robust glucose-lowering capacity and convenient once-weekly dosing, tirzepatide represents a significant advancement in the therapeutic landscape of T2DM.

### 15.2 Orforglipron

Orforglipron is an innovative oral, non-peptide GLP-1 receptor agonist that was assessed in a 26-week, randomized, double-blind Phase II trial involving 383 adults with type 2 diabetes. At daily doses of ≥12 mg, it produced a mean HbA_1_c reduction of 2.10% and weight loss averaging 10.1 kg (7.9%), in contrast to changes of –0.43% HbA_1_c and –2.2 kg weight in the placebo group, and –1.1% HbA_1_c with –3.9 kg weight loss in the dulaglutide-treated arm (141). Adverse events were consistent with GLP-1 therapies, primarily mild-to-moderate gastrointestinal symptoms, while hypoglycemia was uncommon and non severe (142). A subsequent pooled analysis confirmed significantly greater proportions of patients achieving ≥5%, ≥10%, and ≥15% weight loss compared to placebo, along with pronounced improvements in fasting glucose and BMI (141) These strong Phase II results underpinned the Phase III ACHIEVE-1 trial, which demonstrated dose-dependent HbA_1_c reductions of ~1.3–1.6% and ≈7.9% weight loss at 40 weeks, supporting the feasibility of oral GLP-1 therapy as a non-injectable alternative (143).

## 16 CagriSema (semaglutide + cagrilintide)

CagriSema integrates a once-weekly injection of semaglutide (2.4 mg) and cagrilintide (2.4 mg). In a 32-week, randomized Phase II trial involving 92 adults with type 2 diabetes mellitus (baseline HbA_1_c between 7.5% and 10.0%, BMI of 27 kg/m² or more), the combination therapy demonstrated greater effectiveness compared to individual treatments: HbA_1_c levels dropped by 2.2 percentage points, compared to 1.8 points with semaglutide and 0.9 points with cagrilintide (p<0.0001), while weight loss averaged 15.6%, exceeding the 5.1% and 8.1% in the respective single-drug groups (p<0.0001) (144). Notably, continuous glucose monitoring showed a mean time-in-range increase to 88.9% under CagriSema (*vs* 76.2% and 71.7%), and no serious hypoglycemia occurred (144). Mild-to-moderate GI side effects were more common with combination therapy (68%) than monotherapy, but overall tolerability was acceptable (144). These compelling Phase II findings paved the way for Phase III REDEFINE-1/2 trials, where CagriSema achieved up to ~22.7% weight loss in obesity and ~13.7% in T2DM, while maintaining a consistent safety profile (145).

## 17 Antidiabetic agents in development

A new generation of antidiabetic therapeutics is reshaping the landscape of metabolic medicine, emphasizing precision receptor modulation, multi-agonist synergy, and oral bioavailability. Several investigational agents are in advanced stages of clinical development, aiming to extend the physiological reach of incretin-based and immunomodulatory therapies beyond current standards.

### 17.1 LY3502970

Developed by Eli Lilly, LY3502970 is a next-generation GLP-1 receptor modulator characterized by biased agonism. Unlike conventional full agonists, LY3502970 preferentially activates G-protein signaling while minimizing β-arrestin recruitment, a property expected to enhance metabolic efficacy while reducing adverse gastrointestinal effects (146). Its high potency, receptor specificity, and favorable pharmacokinetic profile have enabled oral delivery-an important step toward expanding patient accessibility to incretin therapy (146).

### 17.2 SCO-094

SCOHIA Pharma’s SCO-094 is a dual GIP/GLP-1 receptor agonist designed to leverage the complementary roles of these incretins in glucose regulation (147). The rationale is to combine GIP’s insulinotropic activity with GLP-1’s glucagon suppression and appetite control, potentially yielding superior glycemic and metabolic outcomes compared to single-target agents (147).

### 17.3 Ladarixin

Ladarixin, a CXCR1/2 receptor antagonist developed by Dompé Farmaceutici, represents a distinct immunometabolic approach. By inhibiting interleukin-8-mediated neutrophil signaling, LDX aims to attenuate inflammatory cascades that drive autoimmune β-cell destruction in newly diagnosed type 1 diabetes mellitus (T1DM) (148). However, early clinical data suggest that short-term treatment does not significantly preserve residual β-cell function, underscoring the challenge of halting T1DM progression once immune activation has begun (148).

### 17.4 Danuglipron (PF-06882961)

Pfizer’s Danuglipron is the first non-peptide, orally bioavailable GLP-1 receptor agonist to demonstrate clinically meaningful efficacy in Phase IIb studies. Treatment over 16–32 weeks produced HbA1c reductions of approximately 1.2% and body-weight decreases of 4-9% (149). However, gastrointestinal intolerance (notably nausea and vomiting in up to 73% of participants) and a single confirmed case of drug-induced liver injury led Pfizer to discontinue development in April 2025, highlighting the delicate balance between oral potency and hepatic safety in this drug class (149).

### 17.5 Mazdutide (IBI362)

Co-developed by Innovent Biologics and Eli Lilly, Mazdutide acts as a dual GLP-1 and glucagon receptor agonist. This balanced duality seeks to enhance glycemic control while stimulating energy expenditure through glucagon-driven thermogenesis. Phase II trials in Chinese patients reported HbA_1_c reductions of 1.4-1.7% alongside significant weight loss, supporting ongoing Phase III global development (150).

### 17.6 Retatrutide (LY3437943)

Among the most advanced investigational agents, Eli Lilly’s Retatrutide is a triple agonist that co-targets GLP-1, GIP, and glucagon receptors. By integrating three metabolic axes- insulin secretion, glucagon regulation, and energy expenditure- Retatrutide produces unprecedented efficacy. Phase II data show HbA_1_c reductions of ~2% and body-weight loss of up to 25% at higher doses over 24–48 weeks, outperforming dual incretin agonists such as tirzepatide (151). It now leads the next frontier of poly-agonist therapy in T2DM and obesity.

Together, these investigational compounds represent a paradigm shift- from single-pathway glucose control to multihormonal network modulation. The convergence of receptor bias, poly-agonism, and immune signaling interference defines a new era of antidiabetic drug design, aiming not only to normalize glycemia but to reprogram metabolic homeostasis at its molecular core.

## 18 Limitations and future perspectives

Despite significant advances in elucidating the bidirectional relationship between diabetes, metabolic dysfunction, and infection susceptibility, several fundamental gaps remain. One major limitation lies in the incomplete understanding of how chronic hyperglycemia alters immune cell signaling and tissue-specific defense mechanisms. The molecular underpinnings of impaired neutrophil chemotaxis, macrophage polarization, and T-cell dysfunction in distinct microenvironments- such as diabetic foot ulcers and periodontal tissues- remain incompletely mapped. Furthermore, clinical data are often heterogeneous, constrained by differences in study design, patient demographics, diabetes subtypes, and infection etiologies, making broad extrapolation difficult.

Another growing concern is antimicrobial resistance (AMR). People with diabetes are disproportionately affected by infections caused by multidrug-resistant organisms, yet diabetes-specific antibiotic stewardship frameworks remain underdeveloped. Mechanistic insights are further limited by the predominant reliance on animal models, which often fail to replicate the immunometabolic complexity of human diabetes. Finally, while innovative therapeutic modalities- including GLP-1 receptor agonists, SGLT2 inhibitors, and stem cell-based interventions- show promise, their long-term implications for immune regulation and infection risk remain insufficiently characterized.

Looking forward, precision medicine provides a powerful path to closing these gaps. Integrating genomic, transcriptomic, and microbiome data could enable the identification of predictive biomarkers for infection risk and individualized treatment responses. Similarly, immunometabolic modulation- for instance, through inhibition of the NLRP3 inflammasome-may help to uncouple chronic inflammation from insulin resistance, reshaping the inflammatory-metabolic axis that drives disease progression.

Therapeutic innovation is advancing rapidly. Novel oral incretin agents such as *orforglipron* and multi-agonist compounds like *retatrutide* have the potential to improve metabolic control and patient adherence simultaneously. Complementary preventive strategies should also be prioritized. Expanded vaccination programs targeting respiratory and skin pathogens in diabetic populations, combined with microbiome-targeted interventions (including probiotics, prebiotics, and fecal microbiota transplantation), may help reduce infection burden and systemic inflammation.

In parallel, the rise of digital health technologies opens new frontiers in real-time metabolic and infection surveillance. Artificial intelligence–based analytics could enhance glucose–infection correlation modeling, while telemedicine platforms and digital lifestyle interventions offer scalable solutions for patient engagement and adherence.

Finally, rigorous long-term evaluation of next-generation therapies- such as gene therapy, stem cell transplantation, and metabolic surgery- is essential to define their true capacity to restore immunometabolic homeostasis and mitigate infection vulnerability in diabetes. Together, these directions underscore a strategic shift toward integrative, personalized, and prevention-focused management of diabetes- where infection control becomes an inseparable component of metabolic care.

## 19 Conclusion

Diabetes and metabolic disorders impose a substantial global health burden, exacerbated by complex bidirectional interactions with infectious diseases. The compromised immune function resulting from chronic hyperglycemia, insulin resistance, and inflammation elevates susceptibility to a wide spectrum of infections, including both viral and bacterial pathogens. Bacterial infections such as urinary tract infections, pneumonia, diabetic foot infections, sepsis etc. not only occur more frequently in individuals with diabetes but also tend to present with greater severity and poorer outcomes. At the same time, certain pathogens like *H. pylori*, hepatitis viruses, and SARS-CoV-2, demonstrably worsen metabolic dysregulation, establishing a detrimental cycle that complicates disease management and accelerates progression. Emerging therapeutic strategies such as GLP-1 receptor agonists, SGLT2 inhibitors, nanotechnology-based drug delivery, gene therapy, and stem cell therapy offer promising avenues to modulate both metabolic and immune pathways. Translationally, identifying and targeting shared molecular mediators such as inflammatory cytokines, oxidative stress pathways, and gut microbiota-derived metabolites may yield dual benefits in mitigating infection susceptibility and improving metabolic control. Future research should prioritize longitudinal and interventional studies to delineate causal relationships and evaluate the long-term efficacy and safety of novel therapeutics in diverse populations. Strengthening this translational bridge between immunometabolism and infection biology will be crucial for developing personalized, durable solutions to these intertwined global health challenges.
